# Risk factors assessment for radiographically guided port implantations with forearm access

**DOI:** 10.1371/journal.pone.0259127

**Published:** 2021-10-26

**Authors:** Jonathan Nadjiri, Tobias Geith, Tobias Waggershauser, Stephan Forster, Philipp Paprottka

**Affiliations:** Department of Interventional Radiology, University hospital Klinikum rechts der Isar, School of Medicine, Technical University of Munich, Munich, Germany; Ohio State University Wexner Medical Center Department of Surgery, UNITED STATES

## Abstract

**Introduction:**

Port implantations at the forearm are associated with an increased risk of relevant vein thrombosis. Therefore, with this study we sought to identify the responsible risk factors to improve technical quality of the method.

**Methods:**

This is a retrospective analysis of 313 patients with port implantation at the forearm in 2019. Then, exploratory statistics were conducted comprising Cox-Regression and Kaplan-Meier-Analyses.

**Results:**

Mean age was 60 ± 14 years. 232 (74%) of the patients were female. No early infection was observed. 29 late infections and 57 cases of thrombosis occurred. In only 9% of the patients with thrombosis hospital admission was necessary for treatment. Median interval to the diagnosis of thrombosis was 23 days; inter-quartile-range: 16–75. Mean interval to elective port explantation was 227 ± 128 days. There was no effect of occurrence of thrombosis of the interventionalist, the assistance nor of several technical aspects. However, there was a significantly lower risk of thrombosis for primary implanted port system compared to replacement ports, Hazard-ratio: 0.34 [Confidence interval: 0.172, 0.674], p = 0.002. Age was a significant risk factor for late infections, Hazard-ratio: 3.35 [Confidence interval:1.84, 6.07], p < 0.0001.

**Conclusion:**

The main risk factor for adverse outcome after radiographically guided port implantation at the forearm is the type of the implanted port system. The reason for that might not be the material itself but the experience of a team with a certain port system. Age is a risk factor for late complications.

## Introduction

Cancer is a major cause of morbidity in industrial nations and oncologic treatment is an important aspect in modern heath economics in these countries [1–3]. In many cases systemic chemotherapy is a crucial part in the treatment strategy. Additionally, constant parenteral nutrition or antibody therapy may follow or compliment conventional medication [3–6]. Port catheters play a significant role in oncologic therapy but also in the treatment for other diseases [3]. The most established area for port catheter implantations is the pectoral region either in the surgical variant with preparation of the basilic vein or the interventional variant with needle-based access to the subclavian vein [3]. These techniques have inherent advantages such as short procedure times, short catheter lengths and low rates of thrombosis. But they also have characteristic disadvantages such as risk of pneumothorax or hematothorax and sometimes the need for general anaesthesia [7]. Implantation of a port catheter on the forearm does shift the specific advantages and disadvantages substantially. The risk of severe complications is generally lower, e.g. an iatrogenic pneumothorax is practically excluded. However, due to the long course of the catheter through small veins the risk of an arm vein thrombosis is significantly increased [3]. Besides these attributes individual cosmetic preferences may play a role in the patient’s choice of the location of implantation [8]. Younger women sometimes prefer the forearm port in systemic treatment for breast cancer. Additionally, removing clothes for port access can be facilitated with forearm ports.

The comparability of studies is limited because the occurrence of complications after the implantation of port catheters is heterogeneous. Besides, the evidence of prophylactic administration of anticoagulants to prevent thrombosis after implantation of ports with a forearm access is non-existent [9].

## Methods

### Ethical statement

All procedures performed in studies involving human participants were in accordance with the ethical standards of the institutional and national research committee and with the 1964 Helsinki declaration and its later amendments or comparable ethical standards. This study was approved and patients informed consent was waived by the local ethics committee (Ethikkommission an der Technischen Universität München; Zeichen: 223/20 S).

### Study population

This is a retrospective analysis of all patients with port implantation at the forearm from January to July 2019 in our department.

### Description of port catheter implantation with forearm access

Sterile preparation of the interventionalist and the implantation area. Tightening of the tourniquet. Veinipuncture with a 18 G Braun(Germany) i.v. catheter. In a few cases vein puncture was conducted with ultrasound assistance. Then a hydrophilic terumo 0.035" standard wire was advanced to the right atrium under fluoroscopic guidance. 0.5 ml of Scandicain was administered intracutaneously at the puncture site. The puncture site was augmented to a diameter of 3 mm using a standard scalpel. Then a short 6 F-sheath (Terumo, Japan) was introduced. After this, the port catheter itself was inserted over the wire. Then 9.5 ml of Scandicain was administrated subcutaneously 4 cm distal of the puncture site for the skin inscision. Then the sheath and the wire were removed. Skin inscision was made using a standard scalpel (approx. 2 cm) and the port pocket was formed utilizing blunt preparation. A standard tunneler was used to transfer the catheter end to the port pocket. Then port the reservoir and port catheter were connected and visually tested for leakage. After that, the reservoir is placed deep in the pocket. The skin incision for the port pocket was closed with two to three Donati’s sutures. The puncture site was closed using a simple interrupted stitch. Finally, the port reservoir was punctured percutaneously with a non coring needle and an angiogram was performed to document the correct placement of the catheter tip. Reference is the characteristic contrast of the pulmonary artery.

**Fig 1:**
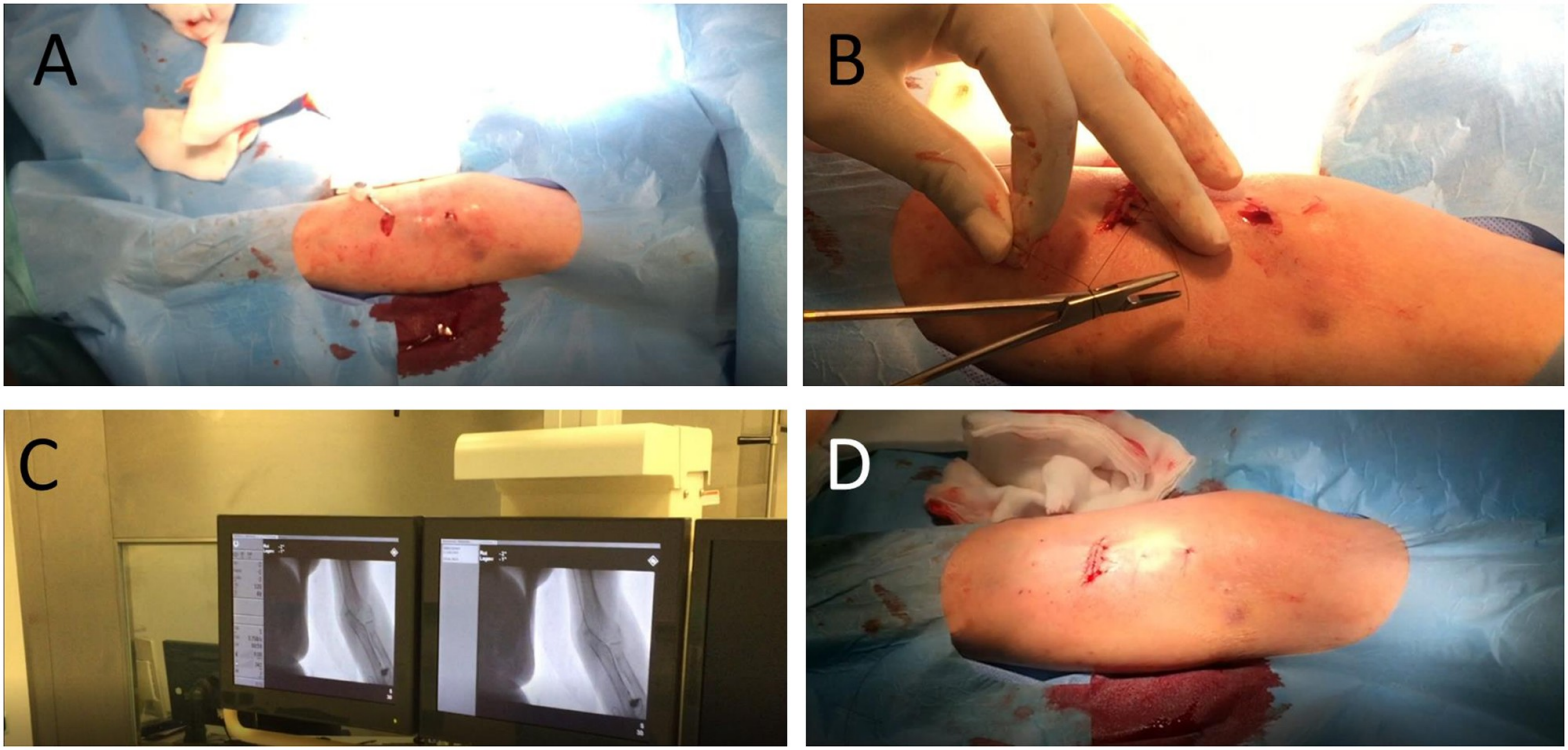
In **A** catheter preparation is shown during the implantation. **B** shows suturing of the access the subcutaneous port chamber. In **C** the radiographic control of the port chamber and the catheter is seen. **D** illustrates the final result after implantation before bandaging.

In the observation interval three types of ports from the company Smiths Medical (USA) were used: Port-A-Cath, P.A.S Port T2 and P.A.S. PORT Elite. All port systems were implanted with a 5.8 F catheter. One catheter was silicon based, the others were made from polyurethane. Key parts of the surgery are provided in Fig 1.

### Endpoints, quality parameters and variables

In this study, occurrence of and interval to thrombosis at the operated arm was considered a primary endpoint. Additionally, the incidence of infection was assessed. The degree of the complication was documented. Early infection was defined as occurrence of inflammation within the first 10 days after implantation. Late infection was defined with clinical signs of inflammation after 10 days.

### Statistics

Exploratory statistics were conducted comprising Cox-Regression and Kaplan-Meier-Analyses. For analysis between survival curves a log rank test was done. Where appropriate, a two-sided t-test was used. The level of significance was adjusted to p = 0.05. All calculations were done using R Project for Statistical Computing with the package “The great truth” [10].

## Results

### Study population

In this study 313 patients were included for analysis. Out of those 232 (74%) were female. Mean age was 60 ± 14 years. More patients’ details are provided in Table 1.

**Table 1:** The most important clinical parameters of the study population.

n = 313	
Age	60 ± 14 years
Female	232 (74%)
Operation room RO16 (vs. RO15)	245 (78%)
Confirmed complications (inkl. late infection during follow up)	86 (27%)
Type of complication (overlap possible)	
Early infection (<10 days)	0
Late infection (>10 days)	29 (9%)
Thrombosis	57 (18%)
Degree of thrombosis	
Outpatient treatment	48 (84%)
No treatment required	3 (5%)
Hospital admission	6 (9%)
Days until diagnosis of thrombosis (Median [IRQ])	23 [16–75] days
Follow-up interval	227 ± 128 days
Access	
Basilic vein	68 (21%)
Brachial vein	140 (44%)
Cephalic vein	103 (32%)

### Events

No early infections were observed. 86 (27%) late infections of the port system were detected. Overall, 57 patients (18%) had a reported thrombosis and/or thrombophlebitis. Out of those, 48 patients (84%) were successfully treated as outpatients. 6 patients with thrombosis (9% of all patients with thrombosis and 3% of all implantations) required hospital admission (no intensive care unit) for further treatment. Median time to diagnosis of thrombosis was 23 days; interquartile range was 16 to 75 days. No explanations were required due to thrombosis neither were negative long-term outcomes associated with thrombosis. Mean follow-up interval was 227 ± 128 days. In Table 2, the subgroup of patients with thrombosis was compared to the subgroup of patients without thrombosis after port implantation.

**Table 2:** Subgroup of patients with thrombosis after port implantation at the forearm with the subgroup of patients without thrombosis after implantation.

	No thrombosis	Thrombosis	p.value
Age	59.8+-14.3	58.3+-13.1	0.44
Female	67 (26.2)	14 (24.6)	0.87
Operateur 1	30 (11.7)	8 (14)	0.89
Operateur 2	86 (33.6)	20 (35.1)	0.98
Operateur 3	33 (12.9)	9 (15.8)	0.84
Operateur 4	47 (18.4)	8 (14)	0.74
Operateur 5	24 (9.38)	1 (1.75)	0.16
Operateur 6	6 (2.34)	0 (0)	0.51
Operateur 7	15 (5.86)	3 (5.26)	0.98
Operateur 8	3 (1.17)	1 (1.75)	0.94
Operateur 9	8 (3.12)	1 (1.75)	0.85
Sterile assistant 1	52 (20.3)	16 (28.1)	0.44
Sterile assistant 2	43 (16.8)	5 (8.77)	0.31
Sterile assistant 3	15 (5.47)	8 (14)	0.073
Sterile assistant 4	34 (13.3)	7 (12.3)	0.98
Sterile assistant 5	35 (13.7)	9 (15.8)	0.92
Sterile assistant 6	41 (16)	6 (10.5)	0.58
Sterile assistant 7	27 (10.5)	4 (7.02)	0.72
Operation room 1	46 (18)	13 (22.8)	0.7
Operation room 2	202 (78.9)	43 (75.4)	0.85
Duration of intervention	13 [9.75,17]	15 [11,18]	0.094
Veneous access			0.47
V. basilica	60 (23.4)	8 (14)	0.3
V. brachialis	114 (44.5)	26 (45.6)	0.99
V. cephalica	80 (31.2)	23 (40.4)	0.42
Right side access	108 (42.2)	28 (49.1)	0.63
Complicated access	39 (15.2)	12 (21.1)	0.32
Sonographically guided access	34 (13.3)	11 (19.3)	0.3

### Thrombosis and thrombosis-free survival

**Fig 2:**
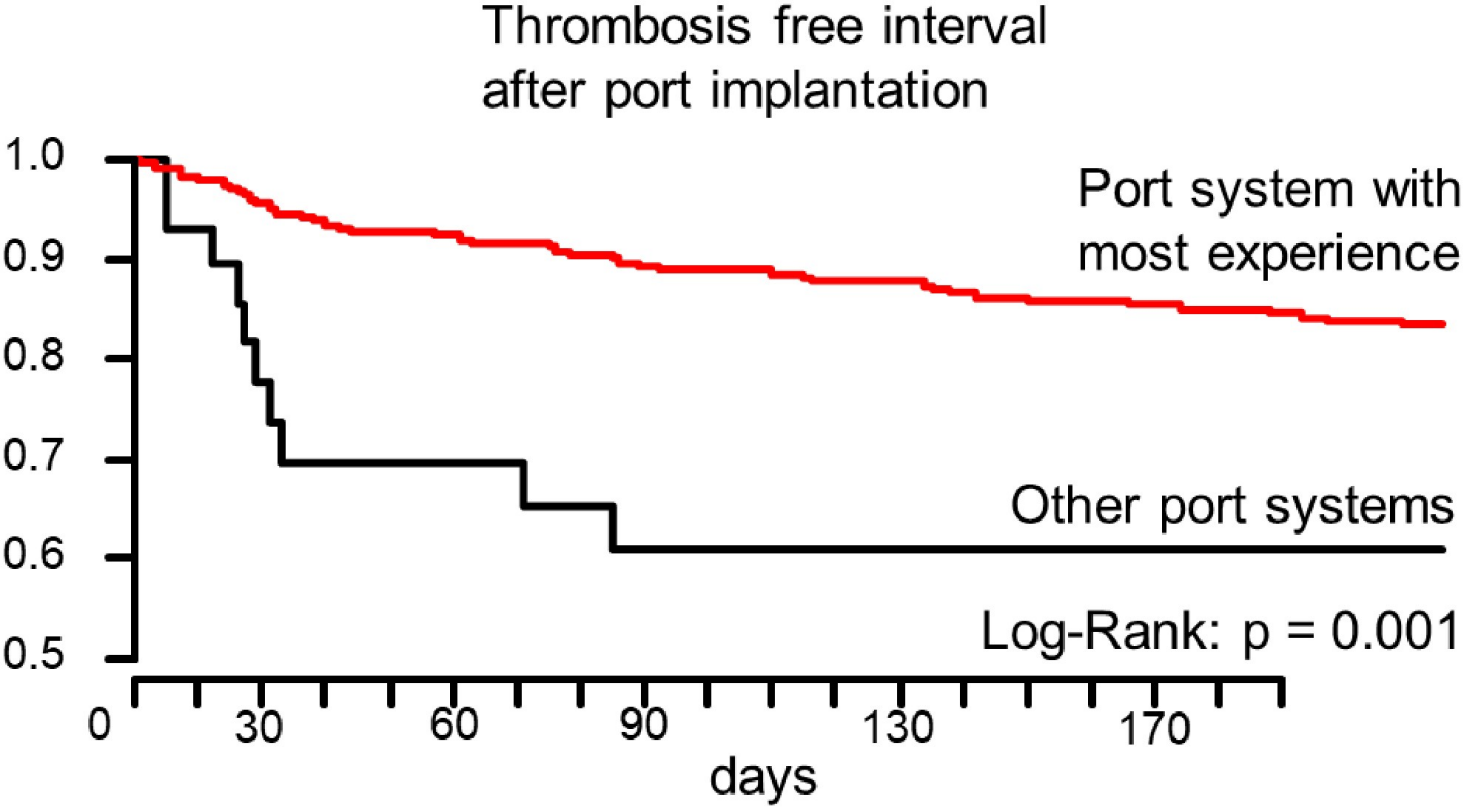
Thrombosis free intervals for the patients with the best-known port system to the team in comparison to other port systems.

We did find best thrombosis free survival for patients with implanted P.A.S. PORT^®^ Elite. That port was used in 284 (90%) of the cases. Implantation of this specific port type led to a significantly decreased risk of thrombosis Hazard-ratio: 0.34 [confidence interval: 0.172, 0.674]; p = 0.002. The difference in thrombosis-free-survival of Kaplan-Meier curves in comparison to the other port types combined was highly significant; Log-Rank: p = 0.001 (Fig 2).

### Exploratory analysis

Variables for exploratory statistics were: interventionalists, sterile assistant, accessed vein, access side(arm), complicated venous access, operating room (OR 1 or OR2), utilisation of sonography for venous access and duration of intervention. Concerning these variables, there was no significant difference for patients with or without thrombosis. Further, thrombosis-free survival was not significantly depending on interventionalist, sterile assistant, accessed vein, side or operating room. Age was a significant risk factor for late infections, Hazard-ratio: 3.35 [confidence interval: 1.84, 6.07], p < 0.0001. More details of the univariate analysis are provided in Table 3.

**Table 3:** Univariate analysis of the most relevant variables on thrombosis free survival.

	No thrombosis	Thrombosis	coef	IQR	OR	z	chi^2	p	C1	z(C)	p(C)
Age	59.8+-14.3	58.3+-13.1	-0.00595	22	0.877[0.589,1.31]	-0.643	0.413	0.52	0.535	0.963	0.34
Female sex	67 (26.2)	14 (24.6)	-0.0158	1	0.984[0.538,1.8]	-0.0512	0.00262	0.96	0.502	-0.052	0.96
Operateur											
1	30 (11.7)	8 (14)	0.209	0	1.23[0.584,2.6]	0.548	0.3	0.58	0.507	-0.326	0.74
2	86 (33.6)	20 (35.1)	-0.0308	1	0.97[0.563,1.67]	-0.111	0.0123	0.91	0.506	0.2	0.84
3	33 (12.9)	9 (15.8)	0.263	0	1.3[0.638,2.65]	0.725	0.526	0.47	0.518	-0.733	0.46
4	47 (18.4)	8 (14)	-0.296	0	0.744[0.352,1.57]	-0.776	0.602	0.44	0.517	0.712	0.48
5	24 (9.38)	1 (1.75)	-1.61	0	0.201[0.0284,1.42]	-1.61	2.58	0.11	0.531	2.4	0.016
6	6 (2.34)	0 (0)	-5.03	0	0.00653[0,49270698]	-0.434	0.188	0.66	0.51	2.32	0.02
7	15 (5.86)	3 (5.26)	-0.0525	0	0.949[0.297,3.03]	-0.0886	0.00785	0.93	0.503	0.2	0.84
8	3 (1.17)	1 (1.75)	0.646	0	1.91[0.264,13.8]	0.641	0.41	0.52	0.504	-0.508	0.61
9	8 (3.12)	1 (1.75)	-0.59	0	0.554[0.0774,3.97]	-0.587	0.345	0.56	0.508	1.07	0.29
Sterile assistant											
1	52 (20.3)	16 (28.1)	0.363	0	1.44[0.806,2.56]	1.23	1.51	0.22	0.531	-1.08	0.28
2	43 (16.8)	5 (8.77)	-0.693	0	0.5[0.2,1.25]	-1.48	2.19	0.14	0.539	2.07	0.038
3	15 (5.86)	8 (14)	0.757	0	2.13[1.01,4.5]	1.99	3.94	0.047	0.534	-1.56	0.12
4	34 (13.3)	7 (12.3)	-0.0578	0	0.944[0.428,2.08]	-0.143	0.0206	0.89	0.505	0.232	0.82
5	35 (13.7)	9 (15.8)	0.127	0	1.14[0.557,2.31]	0.349	0.122	0.73	0.511	-0.432	0.67
6	41 (16)	6 (10.5)	-0.389	0	0.678[0.291,1.58]	-0.902	0.813	0.37	0.518	0.792	0.43
7	27 (10.5)	4 (7.02)	-0.384	0	0.681[0.247,1.88]	-0.741	0.55	0.46	0.514	0.772	0.44
Intervention parameters											
ELite	237 (92.6)	47 (82.5)	-1.08	0	0.34[0.172,0.674]	-3.09	9.55	0.002	0.56	2.32	0.021
T2	8 (3.12)	6 (10.5)	1.44	0	4.21[1.8,9.85]	3.32	11	0.00091	0.543	-2.05	0.041
Port-A-Cath	10 (3.91)	4 (7.02)	0.691	0	1.99[0.722,5.51]	1.33	1.77	0.18	0.519	-1.08	0.28
V. basilica	60 (23.4)	8 (14)	-0.572	0	0.564[0.267,1.19]	-1.5	2.26	0.13	0.543	1.8	0.072
V. brachialis	114 (44.5)	26 (45.6)	0.00773	1	1.01[0.598,1.7]	0.0291	0.000845	0.98	0.501	-0.031	0.98
V. cephalica	80 (31.2)	23 (40.4)	0.394	1	1.48[0.874,2.52]	1.46	2.13	0.14	0.546	-1.41	0.16

The choice of implanted port system was the only relevant factor. Implantation of the ELite port system was associated with better thrombosis free survival while the risk was higher with the T2 port system.

#### Venous access

In 68 cases (21%) the catheter passed the basilic vein, in 140 (44%) the brachial vein and in 103 (32%) cases the cephalic vein. Thrombosis was observed in 11% of the cases with basilic vein access, in 18% with brachial vein access and in 22% with cephalic vein access. In 4% of cephalic vein access, hospital admission was required for further treatment of thrombosis. Hospitalisation for treatment of thrombosis was required in 15% after brachial vein access, in 4% after cephalic vein access and in 12% after basilic vein access.

## Discussion

The main finding of this study is that the implantation of one type of port system is associated with a lower risk of thrombosis after port implantation at the forearm. Further, exploratory analysis showed no relevant influence of variables on the incidence of vein thrombosis after forearm port implantations in our study.

In general, the occurrence of adverse outcomes after port implantations at the forearm are comparable with recent literature [11, 12]. The port systems used in our study were crafted by the same manufacturer and all had the same diameter of the port catheter itself of 5.8F. It can be assumed, that the risk of thrombosis is also associated with an increased diameter of the port catheter and the relation of the port catheter to the accessed vein, respectively. However, in this study all port catheters had the same diameter. According to the personal experience of the authors, port catheters with smaller diameters indeed have a lower risk of vein thrombosis but are prone to material defects in the cubital region. Therefore, catheters with smaller diameters are not implanted at the authors’ department anymore and therefore were not analysed in this study. Since all port systems evaluated in this study were manufactured by the same company, we assume that the differences in the risk of thrombosis are rather a result of the experience of the interventionalists with the respective system than technical reasons of the material itself. Analysis identified a specific port system with a lower risk of complications; but the interpretation has to be that the experience of an interventional team with a certain port system is the actual protective factor. This is further supported by the fact that the port system that has been implanted most often is the one with the lowest complication rate.

The evaluation and comparison of thrombosis after implantation of access ports at the forearm is somewhat complicated since the clinical relevance and also consequence often has not been evaluated on a comparable basis in previous studies. Patients with clinical symptoms of arm vein thrombosis are evaluated carefully at our site. Still, patients treated for thrombophlebitis and thrombosis of the cephalic vein were considered as patients with thrombosis after forearm port implantation with outpatient treatment while the clinical relevance of this complication is low to non-existent. We found a slightly increased risk of thrombosis in the cephalic vein without statistical relevance. The cephalic vein typically has a small diameter with an unfavourable ratio of port catheter diameter compared to vein diameter [13]. Therefore, catheter placement in this vein leads to a relevant reduction of blood flow through supporting thrombosis and thrombophlebitis. Occlusion of the cephalic and basilica vein usually does not cause typical clinical symptoms of arm vein thrombosis such as arm swelling etc. This leads to the conclusion that the cephalic vein is the first choice for the access for forearm port catheter placement. Even with the increased risk of thrombosis associated with access ports at the forearm, the risk of clinically relevant symptoms seems to be more favourable when the catheter is placed in the cephalic vein [14].

In comparison to published data of pectoral ports in the present study no life-threatening conditions such as pneumothorax or hemodynamic relevant bleedings were observed. And evan tough, the incidence of diagnosed thromboses is higher compared to pectoral ports [6]. The rate of required hospital admissions was at an acceptable low rate in this study. Compared with the current literature it can be summarized that complications of any kind are more common at forearm implanted ports compared to pectoral ports but the life-threatening complications are not to be expected and did not occur within our study population [6, 7].

## Conclusion

In our study, the main risk factor for thrombosis after radiographically guided venous access port implantation at the forearm is the type of the implanted port system. The reason for that is most certainly not the material itself but the experience of a team with a specific port system. Increased experience with a certain system is a protective factor. Thromboses of the cephalic vein most often occurred by tendency but had the lowest clinical impact. Therefore, the cephalic vein might be the most eligible access for venous access port implantations at the forearm. Age is a risk factor for late complications.

## Supporting information

S1 Data (CSV)
